# Advancing Prediction of Risk of Intraoperative Massive Blood Transfusion in Liver Transplantation With Machine Learning Models. A Multicenter Retrospective Study

**DOI:** 10.3389/fninf.2022.893452

**Published:** 2022-05-13

**Authors:** Sai Chen, Le-ping Liu, Yong-jun Wang, Xiong-hui Zhou, Hang Dong, Zi-wei Chen, Jiang Wu, Rong Gui, Qin-yu Zhao

**Affiliations:** ^1^Department of Blood Transfusion, The Third Xiangya Hospital of Central South University, Changsha, China; ^2^Department of Blood Transfusion, The Second Xiangya Hospital of Central South University, Changsha, China; ^3^Department of Laboratory Medicine, The Third Xiangya Hospital of Central South University, Changsha, China; ^4^Department of Blood Transfusion, Renji Hospital Affiliated to Shanghai Jiao Tong University, Shanghai, China; ^5^College of Engineering and Computer Science, Australian National University, Canberra, ACT, Australia

**Keywords:** liver transplantation, massive blood transfusion, red cell transfusion, machine learning, prediction model

## Abstract

**Background:**

Liver transplantation surgery is often accompanied by massive blood loss and massive transfusion (MT), while MT can cause many serious complications related to high mortality. Therefore, there is an urgent need for a model that can predict the demand for MT to reduce the waste of blood resources and improve the prognosis of patients.

**Objective:**

To develop a model for predicting intraoperative massive blood transfusion in liver transplantation surgery based on machine learning algorithms.

**Methods:**

A total of 1,239 patients who underwent liver transplantation surgery in three large grade lll-A general hospitals of China from March 2014 to November 2021 were included and analyzed. A total of 1193 cases were randomly divided into the training set (70%) and test set (30%), and 46 cases were prospectively collected as a validation set. The outcome of this study was an intraoperative massive blood transfusion. A total of 27 candidate risk factors were collected, and recursive feature elimination (RFE) was used to select key features based on the Categorical Boosting (CatBoost) model. A total of ten machine learning models were built, among which the three best performing models and the traditional logistic regression (LR) method were prospectively verified in the validation set. The Area Under the Receiver Operating Characteristic Curve (AUROC) was used for model performance evaluation. The Shapley additive explanation value was applied to explain the complex ensemble learning models.

**Results:**

Fifteen key variables were screened out, including age, weight, hemoglobin, platelets, white blood cells count, activated partial thromboplastin time, prothrombin time, thrombin time, direct bilirubin, aspartate aminotransferase, total protein, albumin, globulin, creatinine, urea. Among all algorithms, the predictive performance of the CatBoost model (AUROC: 0.810) was the best. In the prospective validation cohort, LR performed far less well than other algorithms.

**Conclusion:**

A prediction model for massive blood transfusion in liver transplantation surgery was successfully established based on the CatBoost algorithm, and a certain degree of generalization verification is carried out in the validation set. The model may be superior to the traditional LR model and other algorithms, and it can more accurately predict the risk of massive blood transfusions and guide clinical decision-making.

## Introduction

Liver transplantation is generally accepted as the only treatment option for liver diseases such as hepatocellular carcinoma, liver failure, and end-stage liver disease (Jadlowiec and Taner, 2016). Liver transplantation surgery is often accompanied by massive blood loss and massive transfusion (MT; Eghbal et al., 2019; Iyer et al., 2021). In the past, the decision to transfuse red blood cells (RBC) was based on different hemoglobin thresholds set by anesthesiologists (Thai et al., 2020). Affected by many factors, there are certain differences in blood transfusion practices in different institutions. Patient blood management (PBM) is the process of applying evidence-based transfusion guidelines to optimize patient outcomes (Connor et al., 2021). Using hemoglobin concentration as the only trigger for blood transfusion does not fit the modern concept of PBM.

Although MT can save lives in crises, it can cause many serious complications related to high mortality, such as acidosis and blood transfusion-related acute lung injury (TRALI; Muirhead and Weiss, 2017; Meyer et al., 2018; Karim et al., 2020; de Souza et al., 2021). Studies have shown that the need for intraoperative blood transfusion is associated with an increased risk of death after liver transplantation and it is identified as the most important predictor of patient survival (Rana et al., 2013; Cleland et al., 2016; Viguera et al., 2021). A cohort study found that patients who received MT during the perioperative period of liver transplantation had a worse long-term prognosis than non-MT patients, with higher 30-day mortality and complication rates (Tan et al., 2021). Besides, the economic pressure and disease transmission risk brought by MT will further increase.

Machine learning is a subfield of artificial intelligence that allows algorithms to improve their performance on certain tasks based on empirical data (Handelman et al., 2018; Bi et al., 2019; Choi et al., 2020). In recent years, with the development of interdisciplinary, machine learning, as a research hotspot of artificial intelligence, has been widely used in the medical field (Connor, 2019; Sultan et al., 2020; Ding et al., 2021; Hornstein et al., 2021; Huang et al., 2021; Hung et al., 2021; Santos, 2021). In many cases, machine learning algorithms can better describe the complexity and unpredictability of human physiology (Heo et al., 2019). A reliable predictive model can make reasonable use of blood bank resources to avoid waste, besides, it is beneficial to the survival and prognosis of liver transplant patients. Although there have been studies that have constructed predictive models of MT in liver transplantation surgery, they are all based on the traditional logistic regression (LR) method or based on a single-center database. Herein, the new machine learning algorithms are applied to predict MT in liver transplantation based on a multicenter database, aiming to provide a more scientific, reasonable, and effective basis for clinical blood transfusion decision-making and realize the reasonable allocation of blood bank resources.

## Methods

As a retrospective cohort study, we included all patients undergoing liver transplantation in three large grade lll-A general hospitals of China: The Second Xiangya Hospital of Central South University, The Third Xiangya Hospital of Central South University, and Renji Hospital affiliated to Medical College of Shanghai Jiao Tong University from March 2014 to April 2021. Exclude patients: (1) receive preoperative preventive intervention; (2) living donor liver transplantation; (3) orthotopic liver re-transplantation (re-OLT); (4) less than 18 years old; and (5) data loss rate exceeds 20%. Preoperative preventive intervention includes prophylactic platelet transfusion and prophylactic plasma transfusion, which refer to platelet or plasma transfusion in patients without bleeding symptoms before surgery. The blood transfusion strategies implemented by the three hospitals use the restrictive strategies recommended by the current perioperative patient blood management guidelines. A hemoglobin concentration of 7 g/dl was used as a transfusion trigger. When hemoglobin is between 70 g/L and 100 g/L, the clinician will comprehensively judge whether to transfuse or not according to the patient’s age, bleeding volume, bleeding speed, cardiopulmonary function, hypoxia symptoms, and other factors. The goal of blood transfusion is to exceed the threshold and improve the patient’s symptoms. Forty-six adult patients who underwent liver transplantation in the Third Xiangya Hospital of Central South University from May 2021 to November 2021 were collected for prospective verification.

### Variable Selection and Definition

Based on literature search, clinical experience, and expert discussion, 27 candidate risk factors were collected through the electronic medical record system, including patient demographic characteristics, clinical characteristics, diagnosis, and laboratory results. For variables with multiple measurement results, the value closest to the date of the surgery was selected for inclusion in the study. The units of the same indicators were converted into consistent before analysis. The outcome of this study was intraoperative MT, which was defined as the intraoperative transfusion of ≥18 U RBC suspension (1 U RBC suspension equals 200 ml whole blood; Yang et al., 2017).

### Modeling Strategy

**Figure 1:**
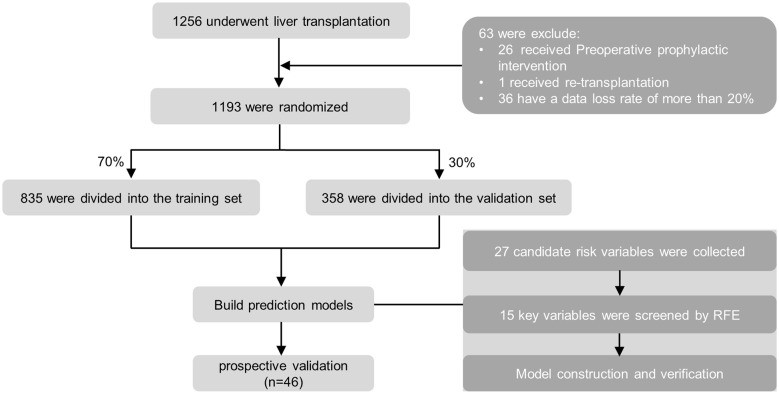
The flowchart of our study.

The data set was randomly divided into the training set (70%, for model development and optimization) and the test set (30%, for model testing). Multiple imputations were used to deal with missing values. The vital features selected by RFE constitute a feature set. Ten machine learning models were established: Categorical Boosting (CatBoost), Extreme Gradient Boosting (XGBoost), Adaptive boosting (AdaBoost), Light Gradient Boosting Machine (LightGBM), Gradient Boosting Decision Tree (GBDT), Random Forest (RF), K-Nearest Neighbors (KNN), Naïve Bayes, Multi-Layer Perceptron (MLP), Support Vector Machines (SVM), LR (Figure 1).

Recursive Feature Elimination (RFE) obtains the optimal combination of variables that can maximize the performance of the model by adding or removing specific feature variables, which was applied to screen key variables. Fifteen key variables were screened out based on the training set, all of which were continuous variables. Further, boxplots were drawn with the key variables to analyze the distribution differences of variables between the two groups. And heatmap was drawn to evaluate the correlation between variables. Then we test the performance of models in an independent test set. In order to test the robustness of the results, we performed 1,000 bootstrap sampling on the test set and evaluated the model separately to generate a confidence interval for the performance of the model.

The existing prediction models are all based on LR analysis, which is very traditional and has limitations. Therefore, after building models, the area under the receiver operating characteristic curve (AUROC), recall rate, sensitivity, and accuracy were used to evaluate and compare the model performance. Three models with better performance were compared with the LR method in the prospective validation set.

### Statistical Analysis

The quantitative data were expressed as mean ± standard or M (P_25_, P_75_) as appropriate, and the qualitative data were expressed as frequency (percentage). The Student’s *t*-test or rank-sum test was used to compare the qualitative data based on whether the variable was normally distributed. The Chi-square test or Fisher’s exact test was used to compare the qualitative data. After modeling, the Shapley additive explanation (SHAP) value was applied to explain the complex ensemble learning model. All analyses were performed using Python (Version 3.7.9) and R (Version 3.6). *P* < 0.05 was considered statistically significant.

## Results

### Clinical Characteristics of the Study Population

A total of 1,193 patients were enrolled in this study, with an average age of 46.15 (11.77) years old, and 210 males (17.60%). According to whether receiving intraoperative MT, they were divided into the MT group [with an average age of 48.96 (9.36) years old, accounting for 15.83% of men] and the non-MT group [with an average age of 45.77 (12.01) years old, accounting for 17.84% of men]. The indexes with statistically significant differences between the two groups were shown in Table 1, including age, clinical diagnosis, portal hypertension, ascites, albumin, activated partial thromboplastin time (APTT), creatinine, hemoglobin, hematocrit, total protein (TP), and urea (*p* < 0.05). All included patients received piggyback liver transplantation.

**Table 1:** Clinical characteristics.

		All Patients (*n* = 1,193)	Non-MT group (*n* = 1,054)	MT group (*n* = 139)	*P*-Value
Age, mean (SD), year		46.15 (11.77)	45.77 (12.01)	48.96 (9.36)	<0.001
Sex, M, n (%)		210 (17.60)	188 (17.84)	22 (15.83)	0.641
Diagnosis, n (%)	Cirrhosis	150 (17.46)	140 (18.49)	10 (9.80)	0.019
	Liver malignant tumor	154 (17.93)	138 (18.23)	16 (15.69)
	Liver failure	83 (9.66)	79 (10.44)	4 (3.92)
	Alcoholic hepatitis	42 (4.89)	33 (4.36)	9 (8.82)
	Viral hepatitis	255 (29.69)	218 (28.80)	37 (36.27)
	Cholestatic liver disease	24 (2.79)	21 (2.77)	3 (2.94)
	Others	151 (17.58)	128 (16.91)	23 (22.55)
Portal hypertension, n (%)		335 (28.08)	280 (26.57)	55 (39.57)	0.002
Hepatic encephalopathy, n (%)		136 (11.40)	117 (11.10)	19 (13.67)	0.609
Ascites, n (%)		385 (32.27)	321 (30.46)	64 (46.04)	<0.001
Weight, mean (SD), kg		64.13 (13.24)	64.38 (13.49)	62.31 (11.10)	0.121
ALB, mean (SD), g/L		34.77 (6.17)	34.96 (6.04)	33.50 (6.86)	0.045
ALT, median [Q1, Q3], U/L		53.85 [26.98, 154.93]	53.85 [27.00, 150.25]	51.85 [26.53, 253.50]	0.729
APTT, mean (SD), s		51.21 (20.13)	50.24 (18.85)	57.25 (26.09)	0.010
AST, median [Q1, Q3], U/L		72.00 [39.00, 197.35]	72.00 [38.40, 183.85]	73.75 [41.22, 283.38]	0.236
CR, median [Q1, Q3], μmol/L		66.90 [55.80, 88.00]	66.00 [55.38, 85.35]	71.00 [58.10, 114.95]	0.010
DBIL, median [Q1, Q3], μmol/L		69.45 [15.97, 231.20]	65.80 [15.30, 237.10]	85.10 [21.95, 200.45]	0.498
GLO, mean (SD), g/L		26.98 (8.77)	27.06 (8.59)	26.48 (9.87)	0.586
HB, mean (SD), g/L		102.47 (25.23)	104.47 (25.20)	89.58 (21.39)	<0.001
HCT, mean (SD), %		30.47 (7.34)	31.14 (7.31)	26.67 (6.36)	<0.001
INR, median [Q1, Q3], U/L		1.63 [1.29, 2.30]	1.63 [1.28, 2.30]	1.58 [1.36, 2.27]	0.700
PLT, median [Q1, Q3], *10^9^/L		69.00 [42.00, 104.00]	71.00 [43.00, 105.00]	63.00 [40.00, 97.00]	0.191
PT, median [Q1, Q3], s		18.95 [15.20, 25.23]	19.00 [15.20, 25.20]	18.10 [15.40, 25.35]	0.881
TBIL, median [Q1, Q3], μmol/L		107.55 [33.82, 380.83]	104.00 [32.30, 384.10]	140.70 [48.05, 336.50]	0.414
FIB, mean (SD), g/L		4.70 (13.77)	3.12 (4.90)	12.10 (30.19)	0.053
TP, median [Q1, Q3], g/L		61.50 [55.00, 68.40]	62.05 [55.30, 68.70]	59.45 [53.27, 65.53]	0.016
TT, median [Q1, Q3], s		19.50 [17.40, 22.20]	19.45 [17.23, 22.10]	19.70 [17.88, 22.92]	0.162
UA, median [Q1, Q3], μmol/L		224.45 [134.40, 332.05]	223.00 [135.05, 330.08]	243.65 [133.20, 357.65]	0.288
UREA, median [Q1, Q3], mmol/L		5.45 [3.87, 8.09]	5.39 [3.82, 7.62]	6.58 [4.07, 10.81]	0.003
WBC, median [Q1, Q3], *10^9^/L		5.22 [3.43, 8.09]	5.28 [3.38, 8.22]	4.95 [3.50, 7.31]	0.318

*Definition of abbreviations: SD, Standard Deviation; ALT, Alanine Aminotransferase; APTT, Activated Partial Thromboplastin Time; AST, Aspartate Aminotransferase; DBIL, Direct Bilirubin; INR, International Standard Ratio; PT, Prothrombin Time; TBIL, Total Bilirubin; TP, Total Protein; TT, Thrombin Time; UA, Uric Acid; WBC, White Blood Cell*.

### Key Features

**Figure 2:**
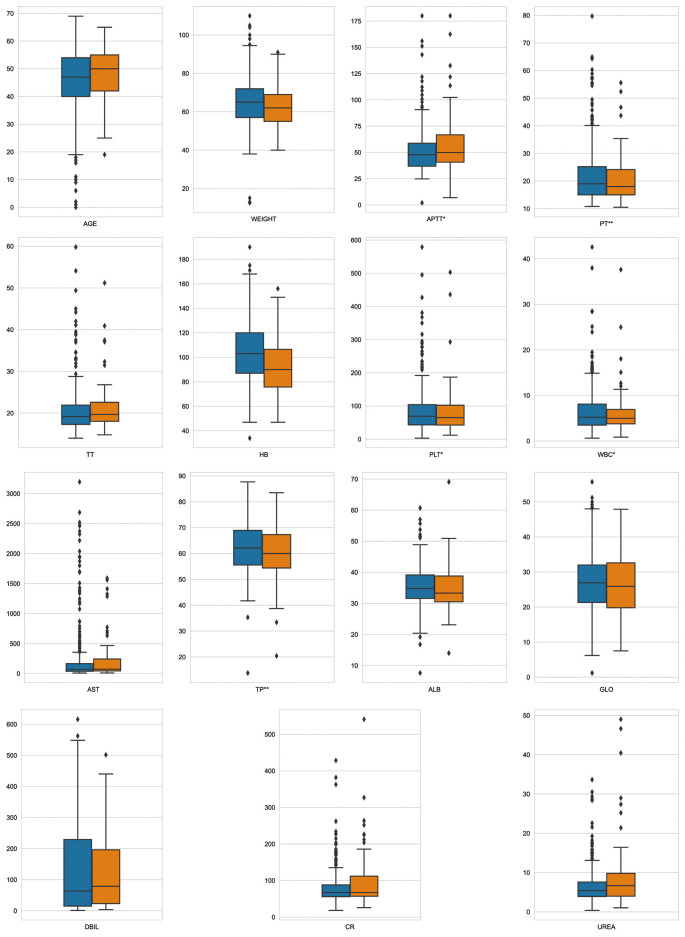
Variable distribution. This figure described the distribution of key variables between groups in the training set. Orange represents MT group, blue represents non-MT group, *p < 0.05, **p < 0.01.

**Figure 3:**
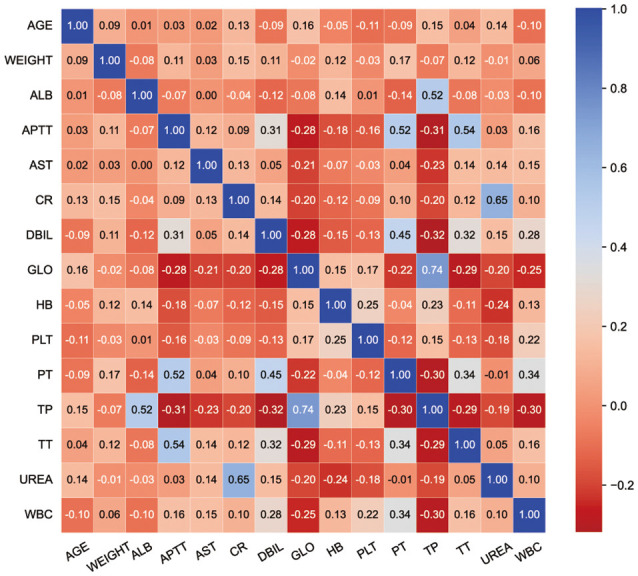
Heatmap. The value in the grid corresponding to the abscissa and ordinate is the correlation value of the two indicators. Corresponding colors and values indicate the degree of relevance.

Fifteen key features on the training set were selected by RFE, including age, weight, hemoglobin, platelets, WBC count, APTT, PT, TT, DBIL, AST, TP, ALB, GLO, creatinine, and urea. Boxplots were used to show the distribution of variables between groups in the training set, from which we can know that patients who is older or whose preoperative hemoglobin lever is lower had a higer risk of receiving intraoperative MT (Figure 2). Pearson correlation coefficients were calculated and a heatmap was used to analyze the correlation between variables. The absolute value of the correlation coefficient ranged from 0 to 1, the greater the absolute value, the stronger the correlation. A positive value indicated positive correlation, while a negative value indicated negative correlation. Generally, the correlation strength of variables was judged by the value range of the following absolute values: 0.0–0.2 (very weak correlation or no correlation), 0.2–0.4 (weak correlation), 0.4–0.6 (medium correlation), 0.6–0.8 (strong correlation), 0.8–1.0 (very strong correlation). For instance, the correlation coefficient between PT and APTT was 0.52, indicating positive and strong correlation, while the correlation coefficient between TP and DBIL was −0.32, indicating negative and weak correlation (Figure 3).

### Prediction Model Performance

**Figure 4:**
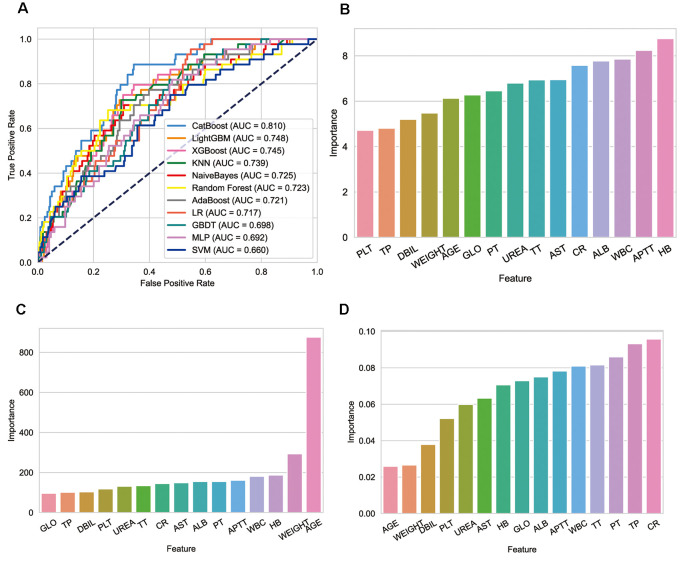
Performance of models and key features. **(A)** Receiver operating characteristic curves for the machine learning models and logistic regression. **(B)** Relative importance of variables included in CatBoost model. **(C)** Relative importance of variables included in LightGBM model. **(D)** Relative importance of variables included in XGBoost model.

Ten machine learning models were constructed. As shown in Table 2, CatBoost performed best in all algorithms (AUROC: 0.81), with a sensitivity of 89% and a specificity of 66% (Figure 4A). The variables included in different models were inconsistent, the relative importance of variables included in CatBoost, LightGBM, and XGBoost were shown in the histogram (Figures 4B–D). Hemoglobin was the most important variable in the CatBoost model, age in the Light GBM model, and Cr in the XG Boost model.

**Table 2:** Prediction model performance.

**Model**	**AUROC**	**Accuracy (%)**	**Youden index**	**Sensitivity (%)**	**Specificity (%)**	**F1 Score**
CatBoost	0.81 (0.75–0.87)	68 (63–73)	0.55	89 (79–98)	66 (60–70)	0.41 (0.32–0.49)
LightGBM	0.75 (0.68–0.82)	70 (65–75)	0.45	76 (62–88)	69 (65–74)	0.38 (0.29–0.48)
XGBoost	0.75 (0.67–0.81)	67 (63–72)	0.46	80 (67–90)	66 (60–71)	0.37 (0.28–0.45)
KNN	0.74 (0.66–0.81)	70 (66–75)	0.43	73 (59–86)	70 (65–75)	0.38 (0.28–0.47)
Naïve Bayes	0.73 (0.64–0.80)	69 (64–74)	0.40	71 (57–83)	69 (63–74)	0.36 (0.26–0.45)
RF	0.72 (0.63–0.80)	74 (69–78)	0.43	68 (53–82)	75 (70–80)	0.39 (0.29–0.49)
AdaBoost	0.72 (0.65–0.80)	62 (58–67)	0.38	78 (65–89)	60 (55–66)	0.34 (0.25–0.41)
LR	0.72 (0.65–0.78)	51 (46–57)	0.41	96 (88–100)	45 (40–51)	0.32 (0.25–0.40)
GBDT	0.70 (0.63–0.77)	56 (52–62)	0.35	82 (70–93)	53 (48–59)	0.32 (0.24–0.39)
MLP	0.69 (0.61–0.76)	56 (50–61)	0.34	82 (69–93)	52 (46–57)	0.31 (0.23–0.38)
SVM	0.66 (0.57–0.74)	55 (50–61)	0.28	75 (62–88)	53 (47–58)	0.29 (0.21–0.37)

*Definition of abbreviations: CatBoost, Categorical Boosting; LightGBM, Light Gradient Boosting Machine; XGBOOST, Extremely Gradient Boosting; KNN, K-Nearest Neighbor; RF, Random Forest; AdaBoost, Adaptive boosting; LR, Logistic Regression; GBDT, Gradient Boosting Decision Tree; MLP, Multi-Layer Perceptron; SVM, Support Vector Machine*.

### Prospective Verification

The three best-performing models (CatBoost, LightGBM, and XGBoost) and the traditional LR method were prospectively verified in the validation set. As shown in Table 3, the sensitivity of CatBoost was 100%, which indicated that the model accurately identified all patients receiving MT in the queue, but the specificity was not the best among several methods. The accuracy of LR was the lowest.

**Table 3:** Prospective verification.

**Model**	**AUROC**	**Accuracy (%)**	**Youden index**	**Sensitivity (%)**	**Specificity (%)**	**F1 Score**
CatBoost	0.75 (0.60–0.88)	63 (50–78)	0.41	100 (100–100)	41 (24–60)	0.67 (0.49–0.80)
LightGBM	0.72 (0.54–0.87)	78 (65–89)	0.51	65 (39–88)	87 (73–97)	0.69 (0.44–0.86)
XGBoost	0.72 (0.54–0.87)	72 (57–85)	0.45	78 (56–95)	69 (50–86)	0.67 (0.47–0.82)
LR	0.61 (0.42–0.77)	59 (43–72)	0.30	89 (71–100)	42 (23–59)	0.61 (0.43–0.75)

### Application of Model

**Figure 5:**
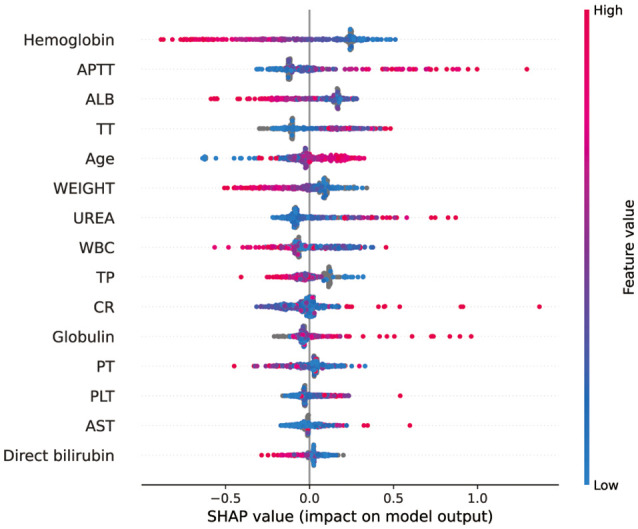
SHAP analysis of the CatBoost model on the validation set. This figure described data from the validation set. Each point represents a sample, and a wide area means a large number of samples are gathered. The color on the right indicates the value of the feature, red indicates that the feature value is high, and blue indicates that the feature value is low.

SHAP (Shapley Additive Explanation) is a “model interpretation” package developed in Python, which can interpret the output of a machine learning model and directly quantify the contribution of each feature to the model’s prediction results. We sorted the included features by calculating the SHAP value (Figure 5). According to the predictive model, the higher the SHAP value of the characteristic, the greater the risk of intraoperative MT. The figure depicted the situation of all samples, including the level of SHAP values of different features and the concentration of SHAP values in the training set. As shown in the figure, the SHAP value of hemoglobin was the highest, which means that hemoglobin concentration contributes the most to the predicted results of the model. When hemoglobin concentration decreased (blue), the model output value was more likely to be bigger (the SHAP value is positive), which means the greater the risk of intraoperative MT.

### Interactive Application

**Figure 6:**
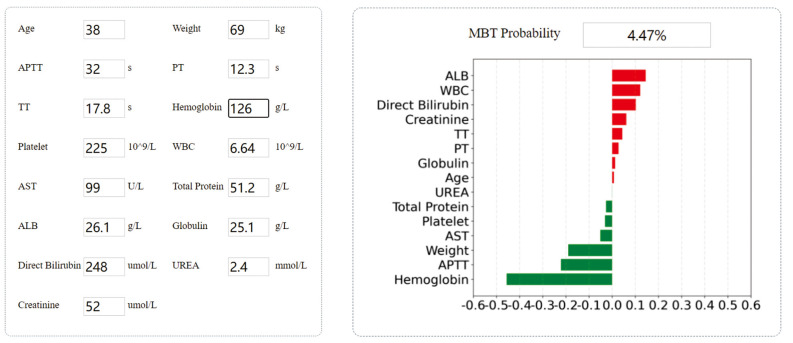
Example of tool usage. Entering the specific input value of each patient to obtain the specific output value. Showing the contribution of each indicator to the prediction result.

We built a user interface^1^ (Figure 6), which allows the anesthesiologist or physician to interact with the model by entering parameter values specific to each patient. The model will predict the probability of intraoperative MT, and doctors can make clinical decisions based on probability.

## Discussion

Predicting the need for MT is directly related to the clinical outcomes and prognosis of patients. This is the first study using machine learning algorithms to build a model to predict the risk of intraoperative MT in patients undergoing liver transplantation. A prediction model with good performance based on CatBoost is successfully constructed, and its performance is better than other machine learning models and LR. The application of the model can reduce the waste of blood products and improve prognosis by predicting the demand for MT and customizing the transfusion scheme. Preoperative hemoglobin, hematocrit, and platelet concentration are often regarded as vital variables for blood transfusion (Stanhiser et al., 2017; Kang et al., 2020). It was found that HCT, FIB, and ALT were important risk factors for MT in liver transplantation patients (124 cases, LR method; Danforth et al., 2020). In the study of Pustavoitau et al. (2017), hemoglobin and platelet concentration were important predictors of MT (203 cases, LR method). Although the variables included in the two studies are not the same, they seem to be connected to each other. The clinical significance of HCT and hemoglobin are similar and can be used for the diagnosis and classification of anemia. Our study also found that these two indicators of MT patients were significantly lower than those of the NMT group. ALT was not included as a key variable in our model, which exists in various cells, especially in hepatocytes. When liver cells are damaged, they will be released into the blood, and the serum ALT level we detected will increase. Patients undergoing liver transplantation often have different degrees of liver lesions, which has been confirmed in our study. ALT in patients with MT and NMT both increased and without significant difference. Therefore, in the prediction of blood transfusion, although ALT will change, it may not be specific and does not perform well in the classifier.

Age, albumin, and creatinine are risk factors for massive hemorrhage in liver transplantation, which has been used by Mccluskey et al. (2006) to develop the McCluskey risk index, guiding MT during surgery. The correlation between the important characteristics involved in the risk index and MT has been verified in two different cohorts. It is worth mentioning that the definitions of MT in the two studies are different. Justo et al. (2021) and the original study defined it as ≥6 U, while Pustavoitau et al. (2020) defined it as ≥10 U. Consistently, these correlations were also found in our study and were screened as key features. In addition, we need to emphasize the reason why our definition of MT is so different from the existing definition. According to the Chinese standard, 1 U RBC suspension equals 200 mL whole blood, which is based on the evidence of clinical transfusion practice in Chinese hospitals, while most of the existing definitions are based on 1 U RBC suspension equals 450–500 ml whole blood (Gurevitz, 2011; Kogutt and Vaught, 2019). Therefore, our definition seems to be significantly far from the current benchmark.

WBC count is generally used for screening blood system diseases and infection, which has been found to be a predictive application in pediatric liver transplantation for MT (Jin et al., 2017). Jin believes that leukocytosis can cause massive bleeding in patients with liver dysfunction. In this study, the model screened the WBC count as an important variable, and in SHAP analysis, its contribution to the prediction result ranked 8/13. We found that the lower the WBC count, the higher the risk of MT, which is inconsistent with other studies. We consider that even if the result is based on the algorithm, it may have no significance in clinical practice, because in fact, the difference in WBC count between groups is not significant, and the mean values are within the normal range. The main non-surgical causes of MT during liver transplantation are coagulation dysfunction caused by coagulation factor deficiency, thrombocytopenia, and hyperfibrinolysis (Villarreal et al., 2019). Therefore, the conventional indicators of coagulation function are of great significance for the prediction of MT. In addition to these indicators, our study also included weight, APTT, PT, AST, TP, globulin, and urea. We believe the reasons why the above studies did not include these indicators may be limited by the relatively small sample size, which reduces the ability to identify important risk factors.

In this research, we built 10 machine learning models. The three better performers are CatBoost, LightGBM, and XGBoost. Among them, Catboost has the best comprehensive performance. CatBoost is a GBDT framework based on trees with fewer parameters, supporting categorical variables, and high accuracy. It solves the problems of gradient bias and prediction shift, thereby reducing the occurrence of overfitting and improving the accuracy and generalization ability of the algorithm (Ambe et al., 2021; Zhang et al., 2021). Compared with XGBoost and LightGBM, CatBoost is an innovative algorithm that embeds automatic processing of categorical features into numerical features. Although LR is easy to understand and implement and is widely used in the study of risk factors for clinical diseases, it has many shortcomings, such as easy under-fitting and low classification accuracy. In this cohort study, compared with LR method-based model, the machine learning model significantly improved the discrimination of risks of intraoperative MT, and had better model predictive capabilities. Regardless of whether it is in the test set or the validation set, machine learning algorithms are always significantly better than LR. However, it should be noted that the hierarchical structure of machine learning algorithm makes it possible that there may not be a linear relationship between the features and the output, such as weight and AST, although they are listed as important features, they did not show significant differences in the comparison between groups.

The SHAP analysis was performed on the model to observe the impact of each feature on the prediction results. At present, many guidelines and clinical practices only use hemoglobin as the basis for blood transfusion. Similarly, our research also found that hemoglobin has the largest contribution to the model’s prediction results, illustrating the importance of hemoglobin in predicting MT. However, importance does not mean uniqueness. In addition to hemoglobin, the role of APTT, ALB, and other variables in the model cannot be ignored, which reminds us that when making blood transfusion decisions, we should comprehensively consider various indicators and not just focus on hemoglobin. It should be noted that some features, such as AST, are not important features for most people, but they may be important for a small group of people. Our figure only represents the overall situation, not everyone’s situation.

CatBoost accurately predicts the risk of MT in the prospective data set, with a sensitivity of 100%. But compared with other methods, the specificity is not the best. We consider this result because the prospective sample size is too small to show the best performance of the model. Although in clinical practice, it seems safer to not miss MT patients. Identifying patients with a high risk of blood transfusion can improve the utilization of blood management during the perioperative period, thereby potentially reducing blood transfusion and its associated risks and costs. In clinical applications, the classification model can be used as a screening tool to quickly and accurately identify patients who require MT. However, we need to recognize that machine learning is a tool that can identify factors that predict a given result, but it cannot prove cause and effect. One of the abilities of machine learning is to help determine new hypotheses for further research. In this case, machine learning has determined that hemoglobin plays a role in prediction, but this does not mean that hemoglobin has a causal role in the need for blood transfusion. More clinical trials may be needed in the future to help understand its causality.

In this study, we proposed a machine learning model to accurately predict the MT need of adult liver transplant patients and a prediction tool that enables clinicians to use to guide clinical decision-making. Its clinical utility lies in that it has specific input and output values for each patient so that precision medicine advice can be generated.

Our study also has some limitations. The first limitation is the inclusion of candidate risk factors. A study found that preoperative blood transfusion was an important risk factor for MT (Danforth et al., 2020). However, due to the large number of missing data on preoperative blood transfusion volume, this factor was not included in the candidate risk factors for this study. In addition, although our study considered the possible effects of different primary diseases on MT, the patient’s disease severity and underlying diseases may also affect intraoperative MT, which was not included in our candidate risk factors. It may have an impact on the ability of our prediction model to identify MT risks. The second limitation is that our study is based on available preoperative indicators. Intraoperative MT is likely to be affected by the transfusion volume of other blood products. Potential intraoperative factors cannot be incorporated into our model for the time being. We hope to overcome this difficulty in the future, which may need to be solved through the interaction of multiple prediction models. Finally, as we discussed above, the number of samples in the prospective validation cohort of the models is limited, which may affect the evaluation of the generalization ability of the model to a certain extent. Multicenter cooperation is expected to make up for this deficiency.

## Conclusion

We have demonstrated that a machine learning algorithm can be used to predict the demand for intraoperative MT in patients undergoing liver transplantation, and we have successfully developed a prediction model based on CatBoost algorithm, which may be superior to the traditional LR method and other algorithms. For better clinical application, we have established an interactive website as a tool, which is the first of its kind known to us. Our team will also be committed to implementing it to bring it into the future clinical workflow.

## Data Availability Statement

The datasets presented in this study can be found in online repositories. The names of the repository/repositories and accession number(s) can be found below: https://data.mendeley.com/datasets/82fty5p7df/1.

## Author Contributions

SC, Q-yZ, and RG designed and performed the study, L-pL, Y-jW, X-hZ, Z-wC, HD, and JW collected the data. Q-yZ processed statistical data. SC drafted the manuscript under the guidance of RG. All authors contributed to the article and approved the submitted version.

## Funding

This work was supported by the National Natural Science Foundation of China (Grant No. 81573091), the Natural Science Foundation of Hunan Province (Grant No. 2021JJ31002), and the Fundamental Research Funds for the Central Universities of Central South University (Grant No. 2021zzts1090).

## Conflict of Interest

The authors declare that the research was conducted in the absence of any commercial or financial relationships that could be construed as a potential conflict of interest. The handling editor ZL declared a past co-authorship with the author Q-yZ.

## Publisher’s Note

All claims expressed in this article are solely those of the authors and do not necessarily represent those of their affiliated organizations, or those of the publisher, the editors and the reviewers. Any product that may be evaluated in this article, or claim that may be made by its manufacturer, is not guaranteed or endorsed by the publisher.

## Footnotes

^1^ http://www.aimedicallab.com/tool/aiml-livermassive.html
