# Erratum: Advancing prediction of risk of intraoperative massive blood transfusion in liver transplantation with machine learning models. A multicenter retrospective study

**DOI:** 10.3389/fninf.2023.1161475

**Published:** 2023-02-21

**Authors:** Frontiers Production Office

**Affiliations:** Frontiers Media SA, Lausanne, Switzerland

**Keywords:** liver transplantation, massive blood transfusion, red cell transfusion, machine learning, prediction model

Due to a technical error, the Conflict of Interest statement had missed stating that the handling editor Zhe Luo declared a past co-authorship with the author Qin-yu Zhao.

A correction has been made to the section “Conflict of Interest”:

“The authors declare that the research was conducted in the absence of any commercial or financial relationships that could be construed as a potential conflict of interest.

The handling editor ZL declared a past co-authorship with the author Q-yZ.”

The publisher apologizes for this mistake. The original article has been updated.

